# Gene therapy with a synthetic adeno-associated viral vector improves audiovestibular phenotypes in *Pjvk*-mutant mice

**DOI:** 10.1172/jci.insight.152941

**Published:** 2022-10-24

**Authors:** Ying-Chang Lu, Yi-Hsiu Tsai, Yen-Huei Chan, Chin-Ju Hu, Chun-Ying Huang, Ru Xiao, Chuan-Jen Hsu, Luk H. Vandenberghe, Chen-Chi Wu, Yen-Fu Cheng

**Affiliations:** 1 Department of Medical Research, Taipei Veterans General Hospital, Taipei, Taiwan.; 2 Department of Otolaryngology, National Taiwan University Hospital, Taipei, Taiwan.; 3 Institute of Brain Science, National Yang Ming Chiao Tung University, Taipei, Taiwan.; 4 Department of Otolaryngology, Taichung Tzu Chi Hospital, Buddhist Tzu Chi Medical Foundation, Taichung, Taiwan.; 5 Program in Speech and Hearing Biosciences and Technology, Harvard Medical School, Boston, Massachusetts, USA.; 6 Grousbeck Gene Therapy Center, Schepens Eye Research Institute and Massachusetts Eye and Ear, Boston, Massachusetts, USA.; 7 Ocular Genomics Institute, Department of Ophthalmology, Harvard Medical School, Boston, Massachusetts, USA.; 8 Harvard Stem Cell Institute, Cambridge, Massachusetts, USA.; 9 Broad Institute of MIT and Harvard, Cambridge, Massachusetts, USA.; 10 Department of Medical Research, National Taiwan University Hospital Hsin-Chu Branch, Hsin-Chu, Taiwan.; 11 School of Medicine, National Yang Ming Chiao Tung University, Taipei, Taiwan.; 12 Department of Otolaryngology–Head and Neck Surgery, Taipei Veterans General Hospital, Taipei, Taiwan.

**Keywords:** Neuroscience, Therapeutics, Gene therapy, Genetic diseases, Neurodegeneration

## Abstract

Recessive *PJVK* mutations that cause a deficiency of pejvakin, a protein expressed in both sensory hair cells and first-order neurons of the inner ear, are an important cause of hereditary hearing impairment. Patients with *PJVK* mutations garner limited benefits from cochlear implantation; thus, alternative biological therapies may be required to address this clinical difficulty. The synthetic adeno-associated viral vector Anc80L65, with its wide tropism and high transduction efficiency in various inner ear cells, may provide a solution. We delivered the *PJVK* transgene to the inner ear of *Pjvk* mutant mice using the synthetic Anc80L65 vector. We observed robust exogenous pejvakin expression in the hair cells and neurons of the cochlea and vestibular organs. Subsequent morphologic and audiologic studies demonstrated significant restoration of spiral ganglion neuron density and hair cells in the cochlea, along with partial recovery of sensorineural hearing impairment. In addition, we observed a recovery of vestibular ganglion neurons and balance function to WT levels. Our study demonstrates the utility of Anc80L65-mediated gene delivery in *Pjvk* mutant mice and provides insights into the potential of gene therapy for *PJVK*-related inner ear deficits.

## Graphical abstract

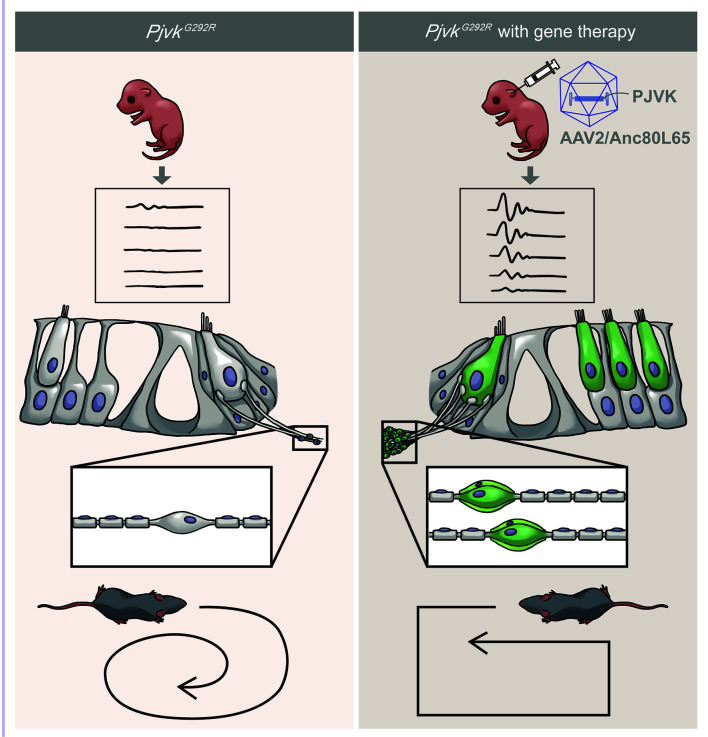

## Introduction

Sensorineural hearing impairment (SNHI) is the most common inherited sensory defect. Permanent SNHI is estimated to occur in approximately 2 out of 1,000 newborns (1). This disorder, including late-onset SNHI, may affect 2% of school-age children (2, 3). Greater than 50% of cases of SNHI in children have genetic causes and are classified as hereditary hearing impairment (HHI) (4). To date, more than 200 genes have been shown to be causally related to HHI (5).

The pathogenesis of SNHI encompasses a wide range of disease mechanisms and can result from damage to any site along the auditory pathway, including hair cells (HCs) or other cochlear cells, spiral ganglion neurons (SGNs), the auditory nerve, or the central auditory pathway. Current treatments for SNHI are mainly tailored according to the severity of the disease. For patients with mild to moderate SNHI, hearing aids are the first-tier treatment. In contrast, for patients with profound SNHI, amplification with hearing aids is usually insufficient because of the severely damaged HCs, and cochlear implantation is the treatment of choice (6). Bypassing the damaged HCs, the cochlear implant directly activates SGNs, transmits auditory signals through auditory nerve fibers and the central neural pathway, and ultimately results in speech understanding in the brain auditory cortex (7). Unfortunately, the outcomes with cochlear implants substantially vary among recipients and are closely related to the underlying pathologies. For instance, our previous study showed that pathogenic variants in the *PJVK* gene (Gene ID: 494513) were associated with unfavorable performance of the cochlear implant (8).

The *PJVK* gene, first identified in 2006 to be linked to a recessive form of nonsyndromic HHI DFNB59 (9), contains 7 exons and encodes the 352 aa protein pejvakin. Pejvakin is expressed in inner hair cells (IHCs), outer hair cells (OHCs), supporting cells, and SGNs in the inner ear (9, 10) and plays a crucial role in action potential transmission, intracellular trafficking, and peroxisomal activity (11, 12). Previous studies in *Pjvk* KO mice demonstrated pathologies involving IHCs, OHCs, and SGNs (12, 13). To address the molecular mechanisms underlying the unfavorable cochlear implant performance in patients with *PJVK* variants, we generated a knock-in mouse model with the *Pjvk* p.G292R (c.874G>A) variant commonly found in the Han Taiwanese population by the CRISPR/Cas9 approach; the *Pjvk* p.G292R mice showed a significant increase in hearing thresholds and impaired balance compared with WT mice (14). Specifically, degeneration of SGNs in histologic analysis was identified, providing insight into the pathogenetic mechanisms underlying the unfavorable cochlear implant performance associated with the *PJVK* variant in our previous clinical observation (8).

The pathological involvement of SGNs, as exemplified by patients with pathogenic *PJVK* variants, represents a limitation of cochlear implantation. Recent advances in gene therapy have suggested therapeutic strategies to address this unmet clinical need. For instance, our previous studies revealed that the synthetic Anc80L65 vector could be used for efficient gene transduction in IHCs, OHCs, and SGNs of neonatal and fetal mice (15, 16) and could restore auditory and vestibular functions simultaneously in mice with certain types of HHI (17). Because the tropism of Anc80L65 in the inner ear parallels the pathologies involved in *PJVK* mutations, in this study, we explored the therapeutic effects of Anc80L65 on hearing and balancing impairments in the *Pjvk* p.G292R mice.

## Results

### Anc80L65-CMV-PJVK shows robust transduction in the neonatal mouse inner ear.

**Figure 1:**
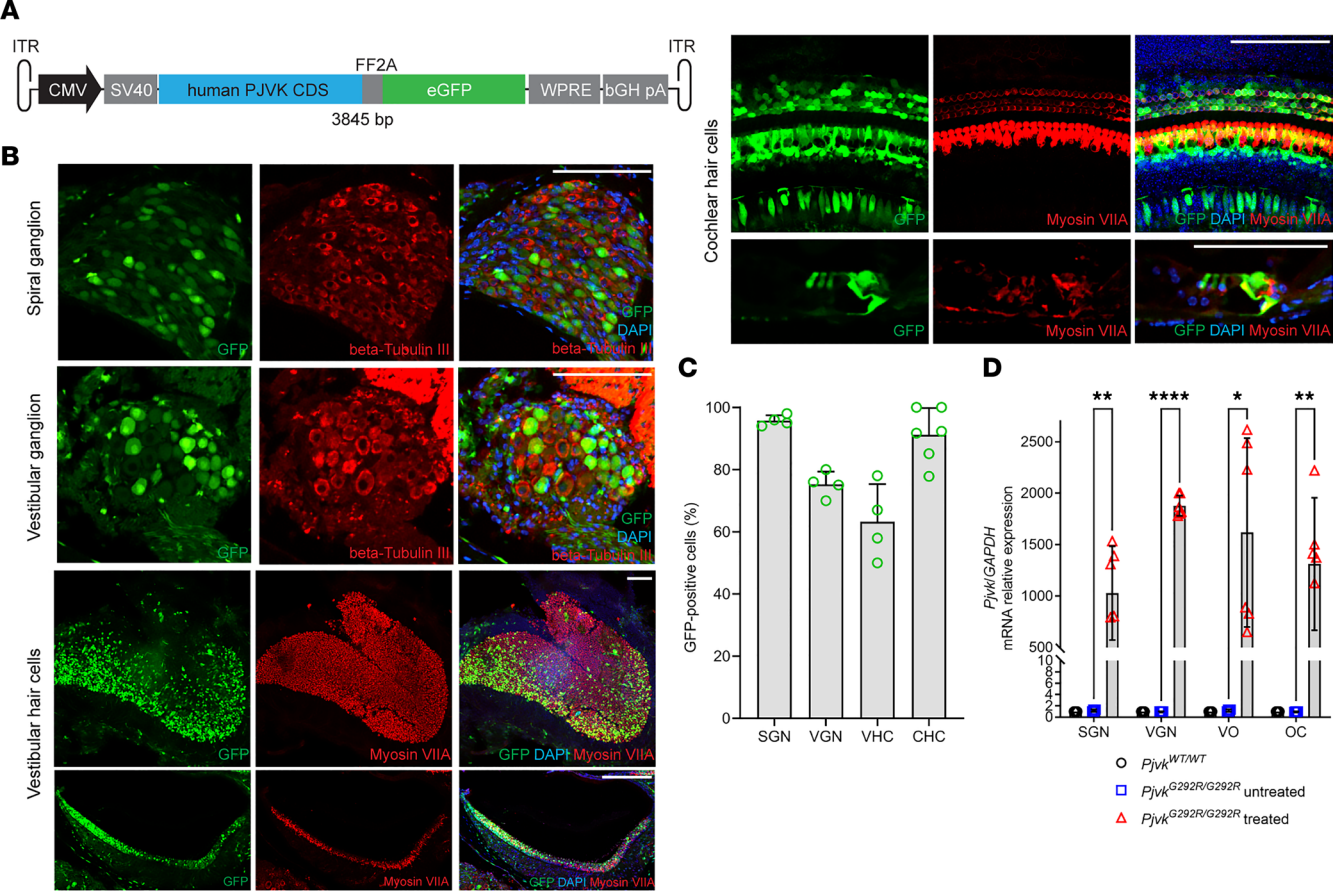
Anc80L65-CMV-PJVK transduction and *PJVK* mRNA expression in the inner ear. P10 *Pjvk^G292R/G292R^* mice injected with Anc80L65-CMV-PJVK at P0–P1 were used to evaluate the transduction efficiency of the virus. (**A**) Schematic diagram of the transgene constructs. The full-length human *PJVK* coding sequence and EGFP sequence were driven by a CMV enhancer and CMV promoter, flanked by AAV2 ITR, and packaged into an Anc80L65 capsid. (**B**) Whole-mount and paraffin-section samples of mouse inner ear organs underwent immunofluorescence staining for β-III tubulin (red) and myosin VIIA (red) to detect neurons and HCs, respectively. Scale bars: 100 μm. (**C**) The transduction efficiency was quantified in the collected parts of P10-treated mice with GFP expression. For SGN and VGN quantification, cells that were double positive for β-III tubulin and GFP were counted; for vestibular HCs (VHCs) and cochlear HCs (CHCs), cells with myosin VIIA and GFP double positivity were counted (*n* = 4 or 6 in each collection part). (**D**) Quantification of human *PJVK* mRNA expression in P10 WT, untreated *Pjvk^G292R/G292R^*, and treated mice. Samples were divided into SGNs, VGNs, VO, and OC and then detected by qPCR (*n* = 4 or 6 in each collection part). Data are shown as the mean ± SD. ****P < 0.0001, **P < 0.01, *P < 0.05, 1-way ANOVA with Tukey post hoc tests.

Anc80L65 is a synthetic capsid facilitating robust transduction efficiency in many cell types in the inner ear without causing significant hearing dysfunction (15, 16, 18, 19). We designed an adeno-associated serotype 2 (AAV2) inverted terminal repeat–flanked (ITR-flanked) construct containing a CMV enhancer and CMV promoter, a full-length human *PJVK* coding sequence, an FF2A peptide, and an EGFP sequence and packaged it into an Anc80L65 capsid, yielding Anc80L65-CMV-PJVK (Figure 1A).

A previous study showed that the *Pjvk^G292R/G292R^* phenotypes, including profound hearing loss and IHC bundle loss, were detected from hearing onset (14); therefore, we decided to treat at the neonatal stage (P0–P1) to stop or slow degeneration. After Anc80L65-CMV-PJVK microinjection via the round window membrane (RWM) approach to *Pjvk^G292R/G292R^* mice at P0–P1, we first examined the transduction efficiency of the viral vector. The mice were sacrificed 10 days after the injection (P10), and the inner ears were collected for whole-mount or paraffinized section immunofluorescence staining (Figure 1, B and C) and qPCR (Figure 1D). The samples were divided into SGNs, vestibular ganglion neurons (VGNs), organs of Corti (OC), and vestibular organs (VO; including the utricle, the saccule, and the anterior, posterior, and horizontal semicircular canals).

Compared with the untreated mice, the treated *Pjvk^G292R/G292R^* mice showed robust GFP expression in SGNs (95.8% ± 1.7% GFP^+^ cells; *n* = 4), VGNs (75.3% ± 4.1% GFP^+^ cells; *n* = 4), vestibular HCs (63.3% ± 12.0% GFP^+^ cells; *n* = 4), and cochlear HCs (91.2% ± 8.6% GFP^+^ cells; *n* = 6) (Figure 1, B and C). Higher human *PJVK* mRNA expression was also observed in all collected parts of the inner ear (Figure 1D). The results indicated that Anc80L65-CMV-PJVK was suitable for Pejvakin inner ear gene therapy.

### Anc80L65-CMV-PJVK rescues Pjvk^G292R/G292R^ SGN density to WT levels.

**Figure 2:**
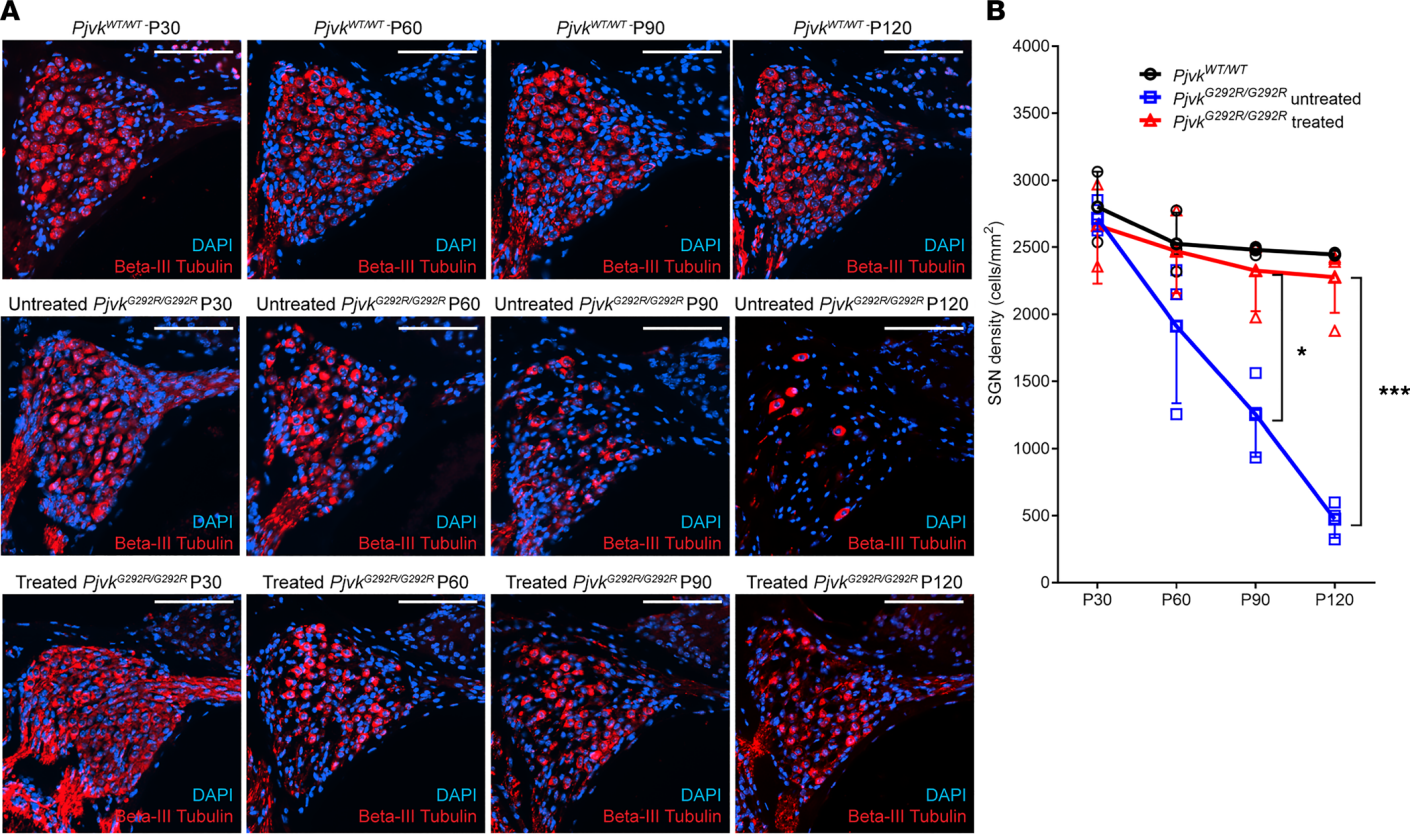
Anc80L65-CMV-PJVK rescues SGNs in *Pjvk^G292R/G292R^* mice. (**A**) Paraffin sections of the inner ears of untreated and treated *Pjvk^G292R/G292R^* mice at P30, P60, P90, and P120. SGNs were stained with anti–β-III tubulin. The WT cochlea is shown as the control. Scale bars: 100 μm. (**B**) Quantification of SGNs (*n* = 3 *Pjvk^WT/WT^* mice, *n* = 4 untreated mice, *n* = 4 treated mice at all time points). Data are shown as the mean ± SD. ***P < 0.001, *P < 0.05, 1-way ANOVA with Tukey post hoc tests.

We first evaluated the therapeutic effect of Anc80L65-CMV-PJVK in SGNs. *Pjvk^G292R/G292R^* mice were injected at P0–P1 with viral vectors through the RWM approach, and inner ear tissue was collected at P30, P60, P90, and P120 for paraffin sectioning and immunofluorescent staining. Untreated *Pjvk^G292R/G292R^* mice and WT samples collected at the same time were used as the negative and positive controls, respectively. The SGN density of the untreated mice was similar to that of the WT mice at P30 but progressively decreased afterward (Figure 2A). Compared with that of the untreated mice, the SGN density of the treated mice was maintained until P120 (untreated *Pjvk^G292R/G292R^* versus treated mice, quantified by β-III tubulin^+^ cells per mm^2^: at P30, 2,731.3 ± 114.0 versus 2,662.9 ± 432.7, *P* > 0.05; at P60, 1,911.4 ± 575.7 versus 2,470.6 ± 312, *P* > 0.05; at P90, 1,386.1 ± 525.4 versus 2,258.5 ± 417.9, *P* < 0.05; and at P120, 473.3 ± 139.7 versus 2,277.0 ± 267.2, *P* < 0.001; *n* = 3 untreated mice, *n* = 4 treated mice at all time points) and showed no significant difference from that of the WT mice at all time points (*n* = 3 *Pjvk^WT/WT^* mice versus *n* = 4 treated mice, *P* > 0.05) (Figure 2B). The results indicated that overexpression of *PJVK* in *Pjvk^G292R/G292R^* mice could significantly rescue the density of SGNs to WT levels. Moreover, this effect could last until at least P120.

### Anc80L65-CMV-PJVK enhances long-term survival and bundle number of OHCs.

**Figure 3:**
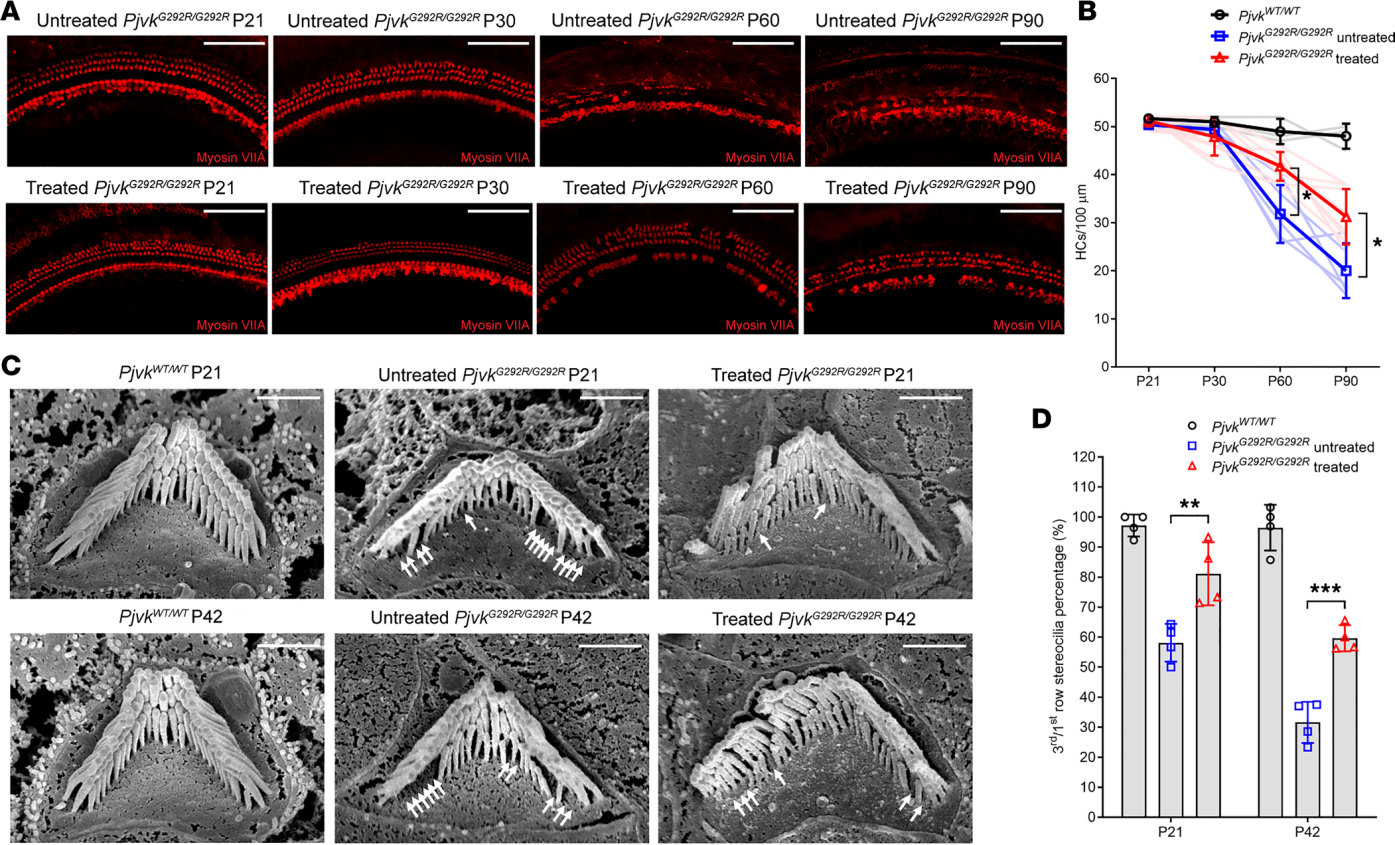
Anc80L65-CMV-PJVK enhances the HC survival rate and OHC bundle number in *Pjvk^G292R/G292R^* mice. (**A**) Whole-mount immunofluorescence at P21, P30, P60, and P90 in untreated (upper panel) and treated *Pjvk^G292R/G292R^* (lower panel) mice. The HCs were stained by myosin VIIA. Scale bars: 100 μm. (**B**) Quantification of HC numbers, presented as HC counts per 100 μm cochlea (*n* = 3 *Pjvk^WT/W^*, *n* = 5 untreated, *n* = 12 treated at all time points). Each line represents 1 animal. Data are shown as the mean ± SD. *P < 0.05 untreated versus treated mice, 1-way ANOVA with Tukey post hoc tests. (**C**) Scanning electron microscopy imaging of HC bundles. The OHC bundles of the 3 groups of mice were detected by scanning electron microscopy at P21 (upper panel) and P42 (lower panel). The white arrows indicate the missing third bundles (*n* = 4 in each group at all time points). Scale bars: 1 μm. (**D**) Quantification of OHC bundle number (*n* = 4 in each group). Data are shown as the mean ± SD. **P < 0.01 untreated versus treated mice at P21; ***P < 0.001 untreated versus treated mice at P42, 1-way ANOVA with Tukey post hoc tests.

Our previous study showed progressive loss of OHCs and their related hair bundles in *Pjvk^G292R/G292R^* mice (14). To test whether *Pjvk* overexpression could reverse these phenotypes in *Pjvk^G292R/G292R^* mice, we collected inner ear tissue at P21, P30, P60, and P90. The HCs were labeled with anti–myosin VIIA and quantified (Figure 3, A and B). We found a significant difference in HC number compared with that of the untreated mice at P60 and P90 (untreated *Pjvk^G292R/G292R^* mice [*n* = 5] versus treated mice [*n* = 12], quantified by myosin VIIA^+^ HCs per 100 μm: at P60, 36.0 ± 9.0 versus 41.8 ± 3.0, *P* < 0.05; at P90, 19.0 ± 4.4 versus 31.3 ± 5.8, *P* < 0.05). Despite the positive effect observed in the treated *Pjvk^G292R/G292R^* mice compared with the untreated mice, the HC number in the treated *Pjvk^G292R/G292R^* mice was still lower than that in the WT mice at P60 and P90 (treated *Pjvk^G292R/G292R^* mice [*n* = 4] versus *Pjvk^WT/WT^* mice [*n* = 3], quantified by myosin VIIA^+^ HCs per 100 μm: at P60, 41.8 ± 3.0 versus 49.0 ± 2.6, *P* < 0.05; at P90, 31.3 ± 5.8 versus 48.0 ± 2.6, *P* < 0.001).

Missing bundles in the third-row OHCs of *Pjvk* mutant mice were described in previous studies (13, 14). To determine whether exogenous *PJVK* could rescue the missing third-row bundles, we counted the third- and first-row bundles and determined the ratio at both P21 and P42 (Figure 3, C and D; the white arrows indicate the missing third-row bundles). Fewer missing third-row bundles were observed in the treated mice than in the untreated *Pjvk^G292R/G292R^* mice at both time points (untreated *Pjvk^G292R/G292R^* versus treated, quantified by the ratio of third- to first-row bundles per OHC: at P21, 58.1 ± 6.3 versus 81.1 ± 10.5, *P* < 0.01; and at P42, 31.6 ± 6.9 versus 59.6 ± 4.3, *P* < 0.001; *n* = 4 untreated mice, *n* = 4 treated mice at all time points). These results indicated that *PJVK* delivery to the inner ear of the *Pjvk^G292R/G292R^* mice could slow the progressive decrease in the loss of both HC numbers and OHC bundles.

### Anc80L65-CMV-PJVK improves auditory functions in Pjvk^G292R/G292R^ mice.

**Figure 4:**
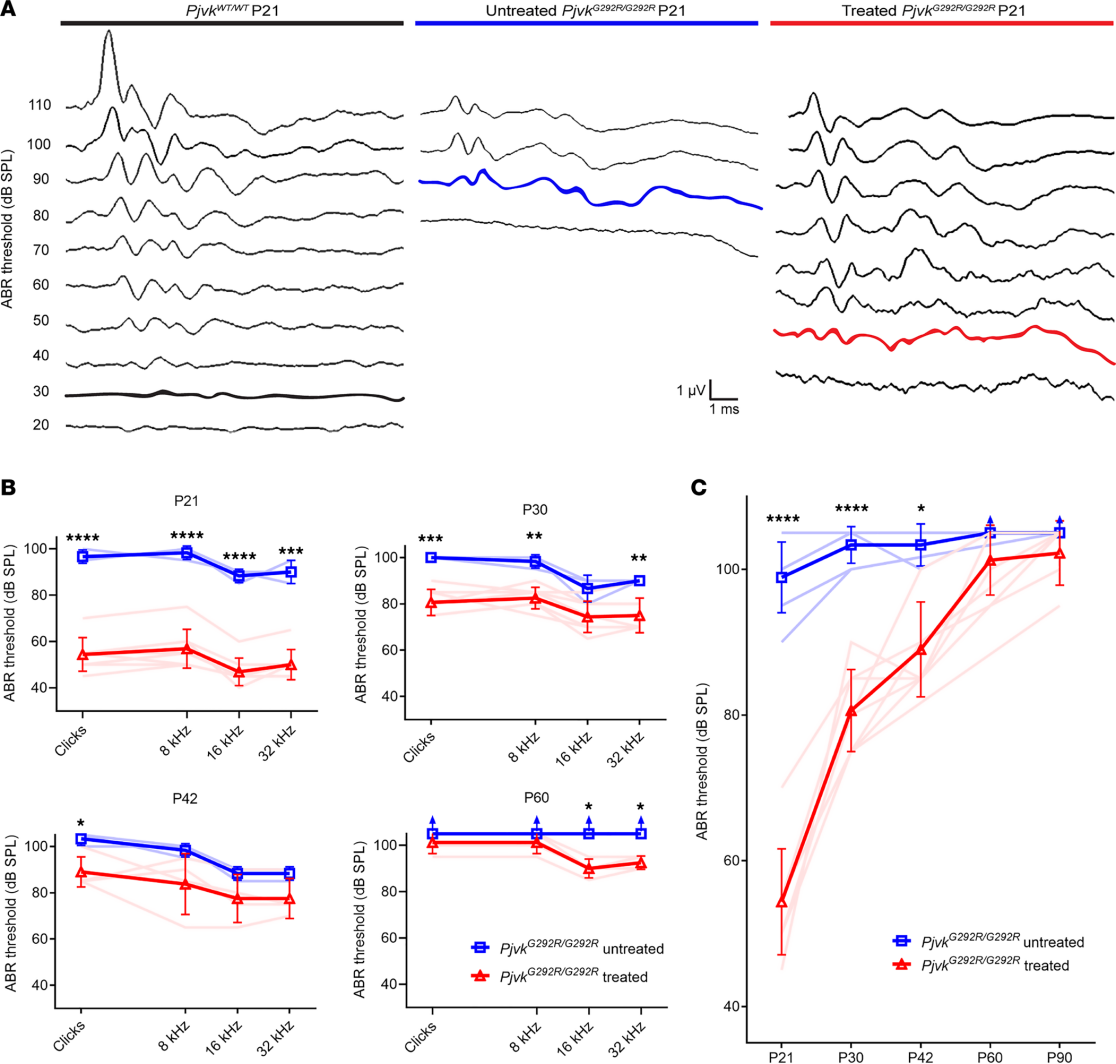
Anc80L65-CMV-PJVK improves auditory function in *Pjvk^G292R/G292R^* mice. (**A**) ABR waveform recorded from P21 mice, using clicks at incrementally increasing sound pressure levels. (**B**) ABR thresholds of the treated WT, *Pjvk^G292R/G292R–^*, and *Pjvk^G292R/G292R^* mice at P21, P30, P42, and P60 (*n* = 3 untreated mice, *n* = 4 or 8 treated mice at all time points). (**C**) ABR threshold for clicks in mice from P21 to P90 (*n* = 5 untreated mice, *n* = 9 treated mice at all time points). Data are shown as the mean ± SD. ****P < 0.0001, ***P = 0.0001, **P < 0.005, *P < 0.05, Student’s *t* test.

To evaluate the therapeutic effect of Anc80L65-CMV-PJVK on auditory functions, we tested the hearing thresholds of untreated and treated *Pjvk^G292R/G292R^* mice by measuring the auditory brainstem response (ABR) at P21 (Figure 4A). The results revealed that gene therapy with Anc80L65-CMV-PJVK could partially restore the mouse hearing threshold at all tested frequencies (at P21: untreated *Pjvk^G292R/G292R^*, 96.7 ± 2.9 dB in clicks, 98.3 ± 2.9 dB at 8 kHz, 88.3 ± 2.9 dB at 16 kHz, 90.0 ± 5.0 dB at 32 kHz; treated *Pjvk^G292R/G292R^*, 54.4 ± 7.3 dB in clicks, 56.9 ± 8.4 dB at 8 kHz, 46.9 ± 5.9 dB at 16 kHz, 50.0 ± 6.5 dB at 32 kHz) (Figure 4B) and resulted in a significant difference (at P21: untreated *Pjvk^G292R/G292R^* versus treated, *P* < 0.0001 at clicks at 8 and 16 kHz; *P* < 0.001 at 32 kHz; *n* = 3 untreated mice, *n* = 8 treated mice at all time points) (Figure 4B).

The ABR waveforms in P21 mice were also analyzed. The absolute and interpeak latencies of ABR waveforms in *Pjvk^G292R/G292R^* mice, recorded at 100 decibel sound pressure level (dBSPL), were all significantly curtailed after the treatment, indicating the alleviation of both cochlear (e.g., absolute latency of wave I) and retrocochlear (e.g., interpeak latencies I–III and III–IV) lesions after gene therapy (Supplemental Figure 1A and Supplemental Table 1; supplemental material available online with this article; https://doi.org/10.1172/jci.insight.152941DS1). We also measured the function of OHCs by using distortion-product otoacoustic emissions (DPOAEs). DPOAE amplitudes were increased at high frequencies (18 and 30 kHz) compared with untreated mice (Supplemental Figure 1B). Despite the nature of progressive hearing deterioration determined by ABR, longer term hearing threshold follow-up at P30, P42, P60, until P90 also showed better results in the treated *Pjvk^G292R/G292R^* mice than in the untreated mice (Figure 4C).

### Anc80L65-CMV-PJVK rescues VGNs and balance functions.

**Figure 5:**
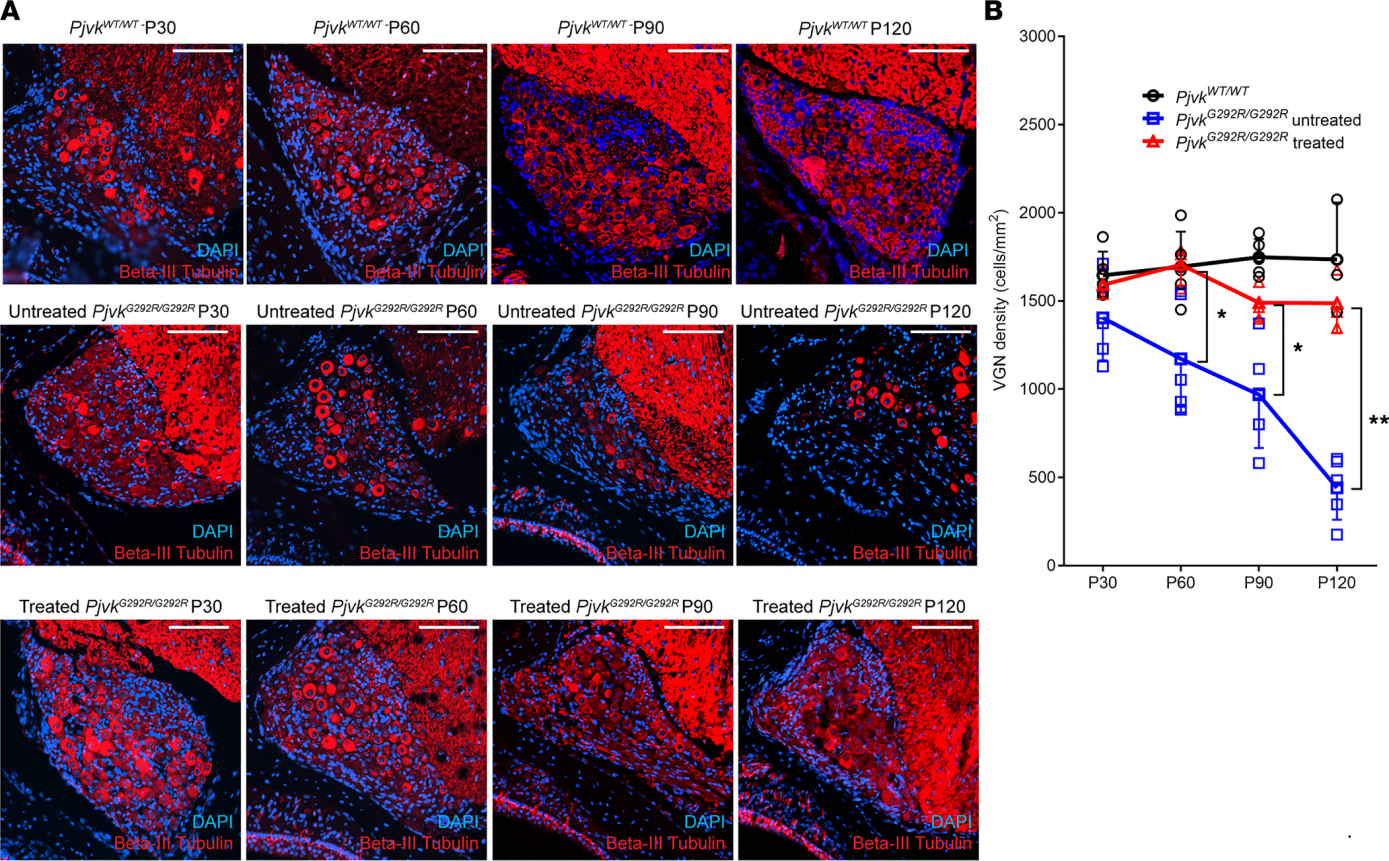
Anc80L65-CMV-PJVK rescues VGN density in *Pjvk^G292R/G292R^* mice. (**A**) Immunofluorescence staining of paraffin sections taken at P30, P60, P90, and P120 from untreated and treated *Pjvk^G292R/G292R^* mice. The WT is shown as a positive control. The VGNs were stained by anti–β-III tubulin. Scale bars: 100 μm. (**B**) Quantification of VGNs (*n* = 5 *Pjvk^WT/WT^* mice, *n* = 5 untreated mice, *n* = 3 treated mice at all time points). Data are shown as the mean ± SD. **P < 0.005, *P < 0.05, 1-way ANOVA with Tukey post hoc tests.

VGN degradation and related balance dysfunction are also major phenotypes of *Pjvk^G292R/G292R^* mice and were previously found to start at P90 in these mice (14). After viral delivery to *Pjvk^G292R/G292R^* mice at P0–P1, the inner ear was sectioned and immunofluorescence stained for VGNs at P30, P60, P90, and P120 (Figure 5A). After quantification of the β-III tubulin^+^ cells, the treated *Pjvk^G292R/G292R^* mice had a higher neuron density than the untreated mice at P60, P90, and P120 (untreated *Pjvk^G292R/G292R^* versus treated, quantified by β-III tubulin^+^ cells per mm^2^: at P60, 1,191.2 ± 329.6 versus 1,706.0 ± 119.0, *P* < 0.05; at P90, 968.6 ± 300.7 versus 1,489.9 ± 106.0, *P* < 0.05; at P120, 441.3 ± 180.3 versus 1,620.1 ± 397.2, *P* < 0.005; *n* = 5 untreated mice, *n* = 3 treated mice at all time points) (Figure 5B) and a similar neuron density as the WT mice (*Pjvk^WT/WT^* mice [*n* = 5] versus treated *Pjvk^G292R/G292R^* mice [*n* = 3], *P* > 0.05 at all observed time points).

**Figure 6:**
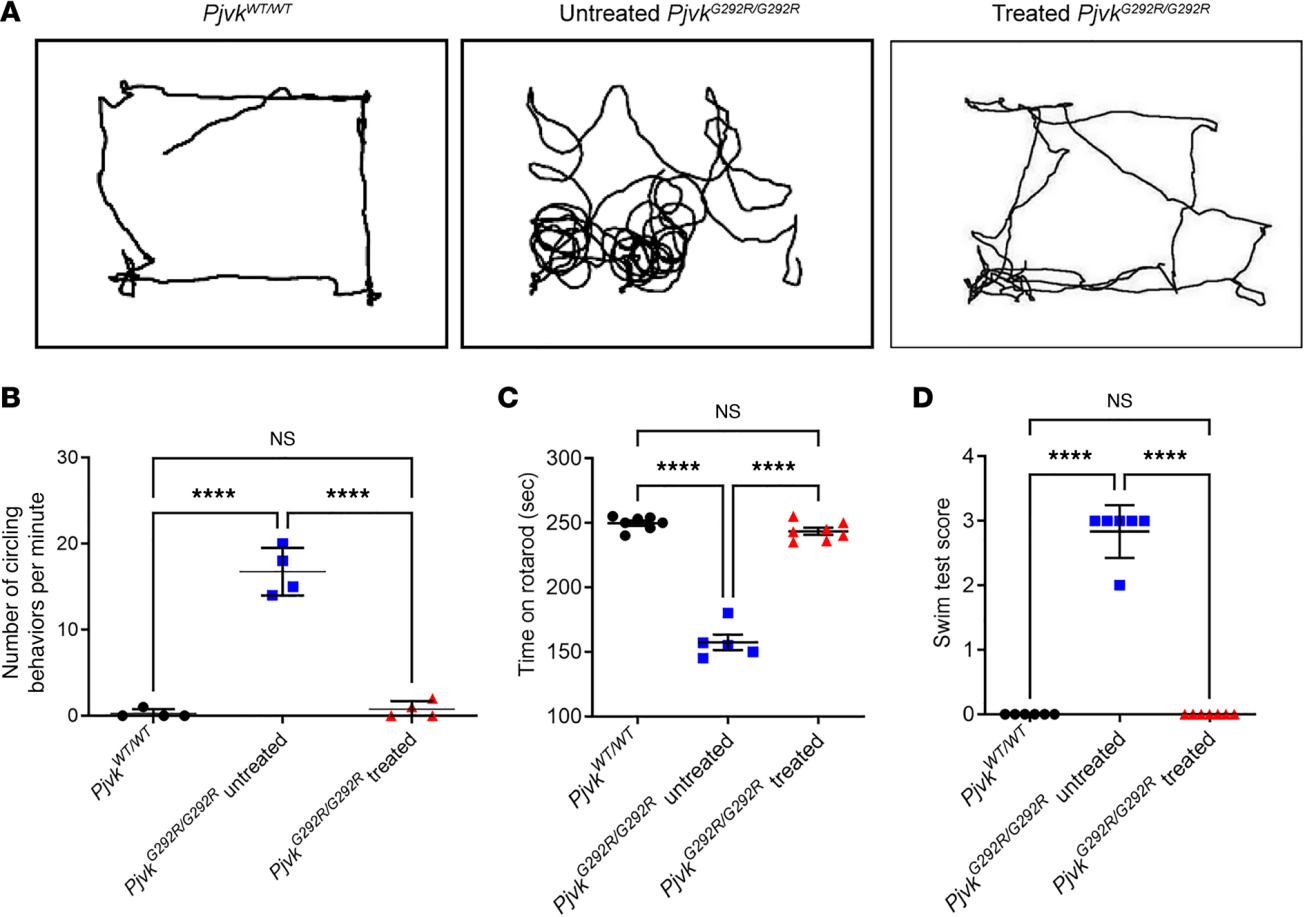
Anc80L65-CMV-PJVK improves balance functions in *Pjvk^G292R/G292R^* mice. (**A**) The open-field test of the WT, untreated *Pjvk^G292R/G292R^*, and treated mice performed at P120. (**B**) Quantification of mouse circling during the 3-minute open-field test (*n* = 4 in each group). (**C**) Quantification of the duration on the rotarod within 250 seconds (*n* = 5 or 7 in each group). (**D**) Quantification of the swimming score (*n* = 6 or 7 in each group). (**B**–**D**) All quantitative data are reported as the mean ± SD. ****P < 0.0001, 1-way ANOVA with Tukey post hoc tests.

To investigate whether overexpression of *PJVK* in the inner ear of *Pjvk^G292R/G292R^* mice would restore vestibule-related balance functions, we performed open-field tests, rotarod tests, and swimming tests on mice at P120 to evaluate balance-related behavior. In the open-field test, we recorded a 3-minute video of the *Pjvk^WT/WT^*, *Pjvk^G292R/G292R^*, and treated *Pjvk^G292R/G292R^* mice to track their circling behavior during walking (Figure 6A and Supplemental Videos 1–3). Similar to the WT mice, the treated mice circled 0 to 2 times per minute, whereas the untreated mice circled 15 to 18 times per minute (rotations per minute: *Pjvk^WT/WT^* mice versus treated *Pjvk^G292R/G292R^* mice, 0.3±0.5 versus 0.8 ± 1, *P* = 0.314; untreated *Pjvk^G292R/G292R^* mice versus treated mice, 16.8 ± 2.8 versus 0.8 ± 1, *P* = 0.0016; untreated *n* = 4, treated *n* = 4) (Figure 6B). Similar results were found in the rotarod test (seconds staying on rotarod: *Pjvk^WT/WT^* mice versus treated *Pjvk^G292R/G292R^* mice, 249.7 ± 5.3 versus 243.4 ± 7.3, *P* = 0.0886; untreated *Pjvk^G292R/G292R^* mice versus treated mice, 157.4 ± 13.5 versus 243.4 ± 7.3, *P* < 0.0001; *n* = 7 *Pjvk^WT/WT^* mice, *n* = 5 untreated mice, *n* = 7 treated mice) (Figure 6C) and swimming test (score for swimming: *Pjvk^WT/WT^* mice versus treated *Pjvk^G292R/G292R^* mice, 0.0 ± 0.0 versus 0.0 ± 0.0; untreated *Pjvk^G292R/G292R^* mice versus treated mice, 2.8 ± 0.4 versus 0.0 ± 0.0, *P* < 0.0001; *n* = 6 *Pjvk^WT/WT^* mice, *n* = 6 untreated mice, *n* = 7 treated mice) (Figure 6D). These results indicated that both *Pjvk^G292R/G292R^* mouse VGNs and the related balance function were rescued by Anc80L65-CMV-PJVK gene therapy.

## Discussion

We previously reported that patients with recessive *PJVK* variants had unfavorable cochlear implant performance (8), which, as shown in our subsequent study in transgenic mice with defective *Pjvk*, might be attributable to degeneration of SGNs (14). In the present study, we further demonstrated that Anc80L65-mediated gene therapy could improve hearing and rescue balance functions in *Pjvk* mutant mice.

Our results showed that Anc80L65 could transduce SGNs and VGNs with high efficiency, and Anc80L65-mediated gene therapy could restore the density of SGNs in *Pjvk* mutant mice. In addition, we also found that myelination of spiral ganglion was disrupted in the *Pjvk* p.G292R mice, and gene therapy with Anc80L65 restored the myelination status of the spiral ganglion (Supplemental Figure 3). Previous studies demonstrated that the density and myelination of SGNs were positively correlated with the neural response to cochlear implant stimulation (20–23). Gene therapies with certain neurotrophic factors, including brain-derived neurotrophic factor and neurotrophin-3, effectively improved SGN density in animal models with SNHI (22–27). In contrast to these nonspecific approaches with neurotrophic therapy for acquired SNHI, the present study demonstrated that the decreased SGN density and myelination defects resulting from specific genetic defects could also be ameliorated by the delivery of the corresponding genes, thus expanding current gene therapy methods for treating SNHI due to different pathogenetic mechanisms. As such, our findings indicate that Anc80L65-mediated gene therapy, when delivered together with the implanted electrode, may serve as a potential modality to augment the function of cochlear implant in patients with defective *PJVK* expression or similar genetic pathologies.

In addition to SGNs, the pathology of *Pjvk*-related SNHI involves IHCs and OHCs (13, 28). Pejvakin is expressed in both IHCs and OHCs (28) and is crucial for the maintenance of stereocilia of IHCs and OHCs (13). Consistent with these lines of evidence, our previous study in *Pjvk* mutant mice identified decreased cochlear microphonic amplitude as a physiological feature and substantial stereocilia loss as a histologic feature, both of which are indicative of pathology in HCs (14). As demonstrated in the present study, both the physiological and histologic changes could be partially rescued by Anc80L65-mediated gene therapy.

Different AAV serotypes display tropism to different cell types in the inner ear. Among the natural AAV serotypes, AAV1–4, AAV7, and AAV8 are efficient for transducing the spiral ligament, spiral limbus, and SGNs; AAV5 is efficient for transducing Claudius cells, sulcus cells, and SGNs; and AAV1–3, AAV5, AAV6, and AAV8 are efficient for transducing IHCs (29–32). Delmaghani et al. (21) reported the use of AAV8 carrying the *Pjvk* transgene in a DFNB59 mouse model, which was found to have variable degrees of hearing loss with degeneration of HCs and spiral ganglions. These researchers found some degree of electrophysiological recovery of auditory phenotypes at P21 after treatment at P3. Although the natural AAV serotypes show inconsistent transduction of SGNs, IHCs, and OHCs, the synthetic Anc80L65 vector was reported to have ideal transduction efficiency in inner ear cell types such as IHCs, OHCs, and SGNs, as shown in our previous studies (16, 18) and in others (15, 17–19, 33–36). We found that gene therapy with Anc80L65 restored the morphology of HCs, increased the density of SGNs, and improved hearing. This feature indicates that the Anc80L65 vector is potentially useful for gene therapy in HHI with pathological involvement of both HCs and SGNs, such as HHI caused by pathogenic *PJVK* variants.

In addition to the auditory system, we observed substantial improvement of vestibular dysfunction in the *Pjvk* mutant mice after Anc80L65-mediated gene therapy (Figures 5 and 6). Vestibular dysfunction has been reported in patients with pathogenic variants in *PJVK* (37). We previously ascribed *Pjvk*-associated vestibular deficits to the degeneration of VGNs because we could not observe pathology in the HCs of VOs in our transgenic mice (14). This hypothesis was substantiated by the findings of the present study, because the restoration of vestibular function concurred with an improved density of VGNs after gene therapy. Notably, vestibular disorders are not uncommon in patients with HHI (38–40). However, compared with auditory symptoms, vestibular disorders have been relatively overlooked by the scientific community thus far, and the genetic basis of vestibular disorders has just started to be understood (40). In addition to this study, a few prior studies documented recovery of balance function after gene therapy in different transgenic mouse models (17, 25, 27, 41, 42), demonstrating the therapeutic potential of gene therapy to address vestibular deficits associated with HHI.

Although *PJVK* gene therapy restores audiovestibular functions in treated mice, we still observed progression of hearing loss until a later stage (P120). Despite the robust expression of pejvakin after the initial delivery, the transgene tends to subside at a later stage (Supplemental Figure 2) and may not be sufficient to reverse the progression of underlying pathology. The restorative effects shown in the vestibular system may be reflected by the relatively late onset of vestibular defects and indicate a relatively longer therapeutic window. More studies are required to improve the sustainability of *PJVK* gene therapy either alone or in combination with cochlear implants in the clinical setting.

In this study, we adopted the RWM approach and delivered viral particles directly into the perilymphatic space of the scala tympani in the cochlea. In addition to the RWM approach, 2 other surgical approaches have been applied to deliver therapeutic agents to the inner ear in animal models, namely, the canalostomy approach, whereby the therapeutic agent is injected through a fenestration in the posterior semicircular canal (18, 43), and the cochleostomy approach, in which a hole near the round window is drilled through the basal part of the cochlea into the cochlear endolymphatic space (29, 44). We observed efficient gene transduction and phenotypic improvement in both the auditory and VOs in *Pjvk* mutant mice after gene therapy with the RMW approach in the present study. This finding has important clinical implications in using gene therapy alone or as an augmenting strategy for cochlear implantation. The RWM approach is currently the most favorable surgical approach for inserting the electrode array of the cochlear implant into the cochlea, because it incurs less surgical trauma to the inner ear than cochleostomy and has a better chance to preserve residual hearing and intricate cochlear structures in patients with cochlear implants (45).

In conclusion, this study revealed that Anc80L65-mediated gene therapy could improve both hearing and balance function in *Pjvk* mutant mice. In particular, our results demonstrated that Anc80L65-mediated gene therapy significantly improved the SGN density in *Pjvk* mutant mice. These results indicate the potential of gene therapy as a standalone treatment as well as in optimizing cochlear implant function in the future.

## Methods

### Animals.

*Pjvk^G292R/G292R^* mice were generated on the C57BL/6J background, which was reported previously (14). The homozygous mice were bred together based on their normal fertility. The WT C57BL/6J mice purchased from BioLASCO Taiwan Co., Ltd., were used as controls.

### Plasmid construction and AAV vector production.

The PJVK-FF2A fragment (1,164 bp) was synthesized and then subcloned into the backbone plasmid pAAV-CMV-RS1-Tag-FF2A-EGFP-WPRE-BGH (provided by the Gene Transfer Vector Core, Massachusetts Eye and Ear Infirmary). Fragment synthesis and subcloning were outsourced and performed by Genscript Biotech Corporation. AAV vectors were produced by using HEK293 cells (Food Industry Research and Development Institute) in hyperflasks with triple plasmid transfection, according to previously described methods (46). Cells were transfected with AAV capsid (Anc80L65), AAV2 ITR-flanked transgene (AAV-CMV-PJVK-FF2A-EGFP-WPRE-bGH), and adenovirus helper plasmids at a ratio of 1:1:2 using polyethylenimine (PEI Max, Polysciences). Three days after transfection, the whole cell culture (i.e., cells and supernatant) was harvested and subjected to lysis, Benzonase treatment, and precipitation in high-salt solution overnight at 4°C. The supernatant was collected after centrifugation and filtration and then concentrated by tangential flow filtration. The full capsids were enriched by iodixanol density-gradient centrifugation. The full capsid-enriched fraction was buffer exchanged and concentrated by running through an Amicon filter with a 100 kDa MW cutoff. The vectors were quantified by qPCR using the TaqMan (Life Technologies) system with primers and probes targeting the promoter sequences of the transgene cassette. Then, the vector purity was assessed by SDS-PAGE. The titer of the virus was 1.51 × 10^12^ GC/mL.

### Mouse experiments.

Neonatal *Pjvk^G292R/G292R^* mice (P0–P1) of either sex were used. All surgical procedures were performed in a clean and dedicated place. The instruments were autoclaved and soaked in 75% ethanol before the surgery. The viral vector was initially aliquoted and stored at –80°C to avoid repeated freezing and thawing. Neonatal mice were anesthetized by hyperthermia on ice for 2 minutes and then placed under a microscope for surgery. For RWM microinjection, a postauricular incision was made to expose the anatomic landmarks of the cochlea. After identification of the anatomic landmarks tympanic ring and stapedial artery, the mice were injected with 1 μL of viral vector within 2 minutes (~8 nL/s) per cochlea. The microcapillaries (Drummond) were held by a micromanipulator (WPI) and controlled by a Micro4 microsyringe pump controller (WPI).

### Immunofluorescence staining.

For whole-mount samples, the mouse inner ears were collected and fixed in 4% paraformaldehyde in PBS at room temperature overnight. After 3 washes with PBS, the inner ears were carefully dissected, and the OCs were divided as previously reported (47). The OCs were permeabilized with 0.25% Triton X-100 and 0.1% paraformaldehyde in PBS at room temperature for 1 hour and then washed 3 times with PBS. After permeabilization, the OCs were blocked by blocking buffer PBT1, which contained 1% BSA, 5% FBS, and 0.1% Triton X-100 in PBS. The OCs were then incubated with the primary Ab anti–myosin VIIA (rabbit, 1:400; Proteus Biosciences) at 4°C overnight. Then, the cells were incubated with Alexa Fluor 568–labeled Abs (1:200; A10042, Invitrogen) at room temperature for 2 hours. After PBS washes, the tissues were mounted using a ProLong Antifade Kit (Molecular Probes). Images were acquired and analyzed by confocal microscopy (Zeiss LSM 880) and ZEN lite software, respectively.

For paraffin sectioning, inner ears were collected and fixed as described above. After decalcification with 5% EDTA in 0.1 M PBS at room temperature over 2 nights, the samples were embedded in paraffin blocks. Then, the blocks were sectioned and immunofluorescence stained with β-tubulin III (rabbit, 1:200; ab18207, Abcam), and/or myelin basic protein (MBP) (rat, 1:100; MAB386, MilliporeSigma), and/or GFP (chicken, 1:1,000; ab13970, Abcam), and secondary Abs.

### qPCR assay.

For identification of exogenous human *PJVK* after viral vector delivery, a pair of appropriate qPCR primers was designed (forward: 5′-GAGAGGCAACCACATCGTGA-3′; reverse: 5′-GGCCTTCACGGCGATAGAAT-3′). After careful separation of the mouse auditory system into the OC, VO, SGNs, and VGNs, human *Pjvk* RNA expression was analyzed by qPCR.

### Ganglion neuron counts.

The cells in sectioned samples with β-III tubulin^+^ and DAPI^+^ cells were counted. For each sample, 3 nonconsecutive sections were selected. The region for calculation was approximately 1 mm^2^ and measured by the open-source image processing software ImageJ (NIH).

### Myelination.

The cells in sectioned samples were triple stained with MBP, GFP, and β-III tubulin Abs. The quantification method for myelination was described previously (48). Briefly, the MBP^+^ myelin sheath was considered intact if it enclosed more than 80% of the outline of the β-III tubulin^+^ cells. Next, the cells with intact myelin sheath were calculated, and the percentage of β-III tubulin^+^ SGNs with intact MBP^+^ myelin sheaths in each group were quantified. For each sample, 3 nonconsecutive sections were selected. The region for calculation was approximately 1 mm^2^ and measured with ImageJ (NIH).

### HC counts.

OC cells that were myosin VIIA^+^ and DAPI^+^ were counted. For each sample, 5 random confocal images were acquired. There are approximately 520–540 HCs/100 μm OC in WT mice.

### OHC bundle count.

Scanning electron microscopy images were used for OHC bundle counting. We randomly chose 5 OHCs per cochlea for quantification, and the total number of cochleae sampled for quantification was 4 per ear group: *Pjvk^WT/WT^*, treated, and treated *Pjvk^G292R/G292R^*. There are 3 rows of bundles per OHC, with 28 to approximately 32 bundles per row, which varies among turns in the cochlea. After calculating the numbers of bundles in the first and third rows of identical OHCs, we calculated the ratio of third and first bundles per OHC to quantify the OHC bundle loss. The detailed quantification method was described previously (13).

### ABR and DPOAE measurement.

Both ABR and DPOAE tests (Supplemental Figure 1B) were performed in an anechoic chamber to avoid background noise. For ABR measurement, click sounds (4–8 kHz) and 8, 16, and 32 kHz tone bursts at various intensities were generated to evoke ABRs (Smart EP 3.90; Intelligent Hearing Systems). For DPOAE recording, 2 speakers presenting 2 primary tones (f1 and f2) at a frequency ratio of 1.2 were coupled to a microphone and introduced into the ear canal of mice; the pure tone amplitudes were fixed at 65 dBSPL for L1 and 55 dBSPL for L2 (Intelligent Hearing System, SmartOAE Program, Intelligent Hearing Systems Corp.).

### Scanning electron microscopy.

For observation of bundle morphology, fresh inner ears were collected and prepared for scanning electron microscopy samples as described previously (49). Samples were subjected to critical-point drying using a Samdri PVT-3D and coated with gold-palladium. Imaging was performed using a JEOL JSM-7600F scanning electron microscope. OHCs were selected from the apical to middle regions of the cochlear spiral.

### Vestibular balance function tests.

Balance function tests were performed according to methods described previously (14). Briefly, in the open-field test, the mice moved freely in an enclosure area, and their movement was recorded with a camera for 3 minutes. The number of times each mouse circled during the 3-minute recording was calculated and quantified. In the rotarod test, a rotarod apparatus with an accelerating program (4–40 rpm over 250 seconds) was used. Three trials per test and an appropriate break time between trials were performed during the test day. The latency to fall off the rotarod was recorded. In the swimming test, the mice were placed in a plastic chamber filled with water, which forced them to swim. The swimming behavior of the mice was scored on the basis of the following criteria: swimming normally for more than 15 seconds: score = 0; swimming poorly and drowning immediately without rescue, score = 3.

### Availability of data and materials.

All data are available in the main text or the supplemental materials.

### Statistics.

Data are presented as the mean ± SD unless otherwise indicated. All values were analyzed by Student’s unpaired *t* test or 1-way ANOVA with Tukey post hoc tests, depending on the experimental groups. Differences with a *P* value of less than 0.05 were considered statistically significant. All analyses were performed using GraphPad Prism, version 9.0.0, for Windows (GraphPad Software).

### Study approval.

All animal experiments were performed according to animal welfare guidelines and approved by the IACUC of the National Taiwan University College of Medicine (approval no. 20200082) and by Taipei Veterans General Hospital (approval no. 2018-148 and 2019-002).

## Author contributions

YCL and YHT contributed equally to this article and are listed based on the alphabetic order of the surname. YFC, CCW, and YCL designed the study. YCL, YHT, YHC, and CJ Hu performed experiments. YHT prepared figures and analyzed data. RX, CJ Hsu, LHV, CCW, and YFC provided reagents, materials, and the mouse model. YFC, CCW, YCL, and YHT wrote the manuscript. CYH, RX, and LHV reviewed and edited the manuscript. All the authors agreed to submit the final manuscript.

## Supplementary Material

Supplemental data

Supplemental video 1

Supplemental video 2

Supplemental video 3

## Version 1 10/24/2022

Electronic publication
