# Supplementary material for: Gene therapy with a synthetic adeno-associated viral vector improves audiovestibular phenotypes in Pjvk-mutant mice
Source: JCI Insight. 2022 Oct 24;7(20):e152941. doi: 10.1172/jci.insight.152941 (PMC9714786; doi:10.1172/jci.insight.152941)
Supplement: Supplemental data [file jciinsight-7-152941-s233.pdf]

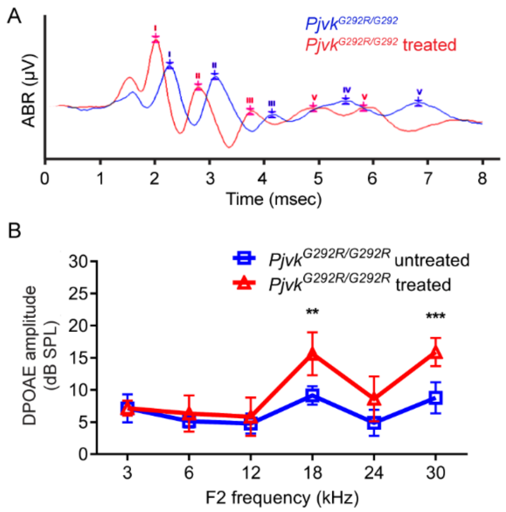

Fig. S1. The ABR waveform and DPOAE levels of ***Pjvk*** $^{G292R/G292R}$ **and treated** ***Pjvk*** $^{G292R/G292R}$ **mice.**

(A) Superimposed ABR traces (click stimuli) at 100 dBSPL. (B) The mean values of DPOAE amplitude in P21 mice (untreated N=11, treated N=9). Data are shown as the mean ± SD. **, p<0.01; ***, p<0.0001. Data are shown as the mean ± SD. (*** P=0.0001, ** P<0.005, Student’s t test).

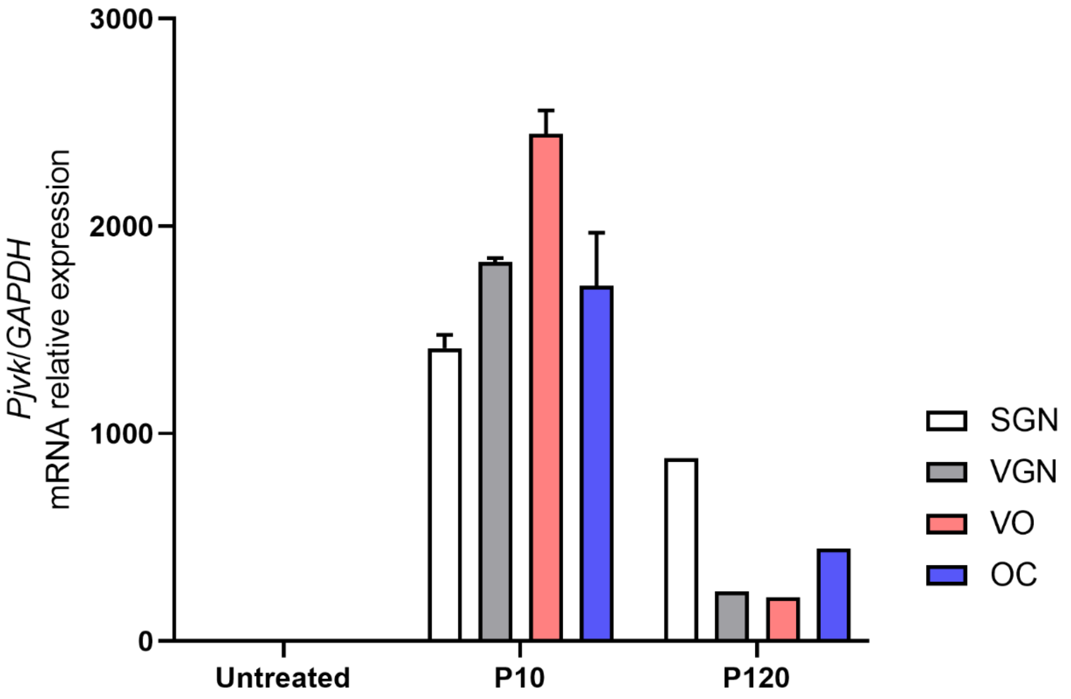

Fig. S2. Long-term *PJVK* mRNA expression in the inner ear organs after Anc80L65-CMV-PJVK neonatal injection.

Quantification of human *PJVK* mRNA expression in untreated and treated $Pjvk^{G292R/G292R}$ mice at different time points. P10 and P120 $Pjvk^{G292R/G292R}$ mice injected with Anc80L65-CMV-PJVK at P0-1 were used to evaluate long-term PJVK expression. Samples were divided into SGNs, VGNs, VO, and OC, and *PJVK* mRNA expression was detected by qPCR (N=5 in the P10 group, N=1 in the P120 group). Data are shown as the mean ± SD. SGN: spiral ganglion neuron, VGN: vestibular ganglion neuron, VHC: vestibular hair cells, CHC: cochlear hair cells, VO: vestibular organs, OC: organ of Corti.

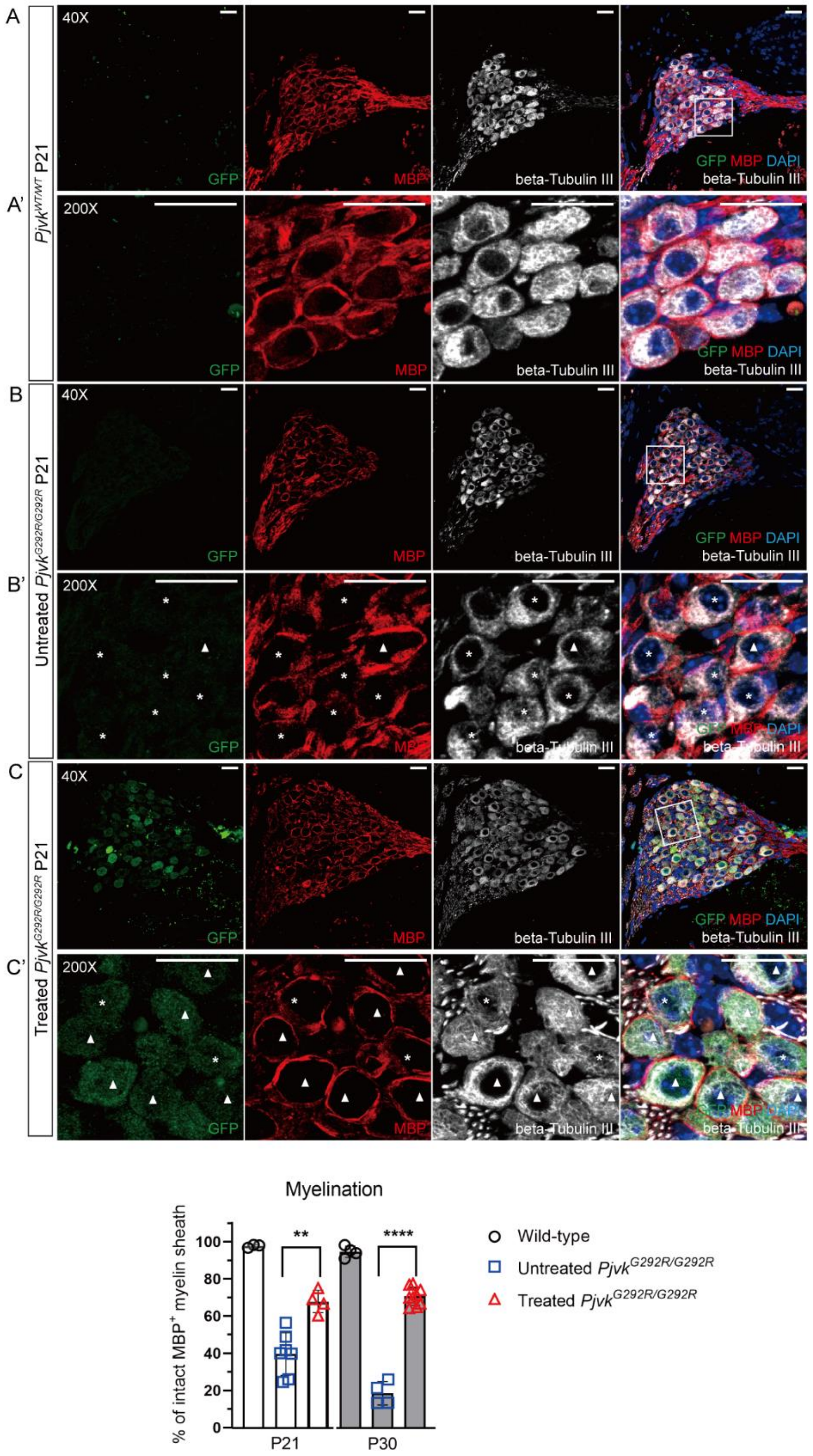

Fig. S3. Anc80L65-CMV-PJVK rescues SGN demyelination in *Pjvk*$^{G292R/G292R}$ mice.

Immunofluorescence staining of SGN paraffin sections taken at P21 from wild-type (A),

untreated (B), and treated (C) *Pjvk*$^{G292R/G292R}$ SGNs. The SGNs were stained with an anti-MBP antibody for the myelination marker and an anti-beta-III tubulin antibody for the neuronal marker. (A-A') Wild-type SGN is shown as a positive control. Most neurons are enveloped by intact MBP+ myelin sheaths. (B-B') Untreated *Pjvk*$^{G292R/G292R}$ mice show relatively normal neuron morphology (beta-III tubulin^{+}) but abnormal myelination (MBP^{+}) in the SGN at P21. Asterisks indicate that the SGNs are partially or not enclosed by myelin sheaths. (C-C') After Anc80L65-CMV-PJVK injection, the MBP and GFP double-positive SGNs in *Pjvk*$^{G292R/G292R}$ mice are represented by arrows. (D) Quantification of the percentage of beta-III tubulin^{+} SGNs with intact MBP^{+} myelin sheaths in each group (P21, *Pjvk*$^{WT/WT}$ N=3, untreated *Pjvk*$^{G292R/G292R}$ N=7, and treated *Pjvk*$^{G292R/G292R}$ N=3; P30, *Pjvk*$^{WT/WT}$ N=4, untreated *Pjvk*$^{G292R/G292R}$ N=4, and treated *Pjvk*$^{G292R/G292R}$ N=9). The MBP^{+} myelin sheath was considered intact if it enclosed more than 80% of the outline of the beta-III tubulin^{+} cells. Scale bars = 20 µm in A-C and 20 µm in A'-C'. Data are shown as the mean ±SD. (** P<0.01 untreated vs. treated mice at P21, ****, P<0.0001 untreated vs. treated mice at P30, one-way ANOVA with Tukey post hoc tests).

Latencies (msec)			
ABR peak	*Pjvk*$^{G292R/G292R}$ (N=10)	Treated *Pjvk*$^{G292R/G292R}$ (N=6)	*P* value
I	2.24±0.05	2.04±0.03	<0.0001
III	4.18±0.11	3.79±0.05	<0.0001
V	6.60±0.17	5.89±0.10	<0.0001
I-III	1.95±0.12	1.75±0.05	0.0018
III-V	2.41±0.14	2.10±0.05	0.0001
I-V	4.36±0.18	3.85±0.09	<0.0001

Statistical tests were Student's t test.

Table S1. Absolute and interpeak latencies of ABR waves of *Pjvk*$^{G292R/G292R}$ and treated *Pjvk*$^{G292R/G292R}$ mice.
